# 4-Chloro-1-iodo-2-nitro­benzene

**DOI:** 10.1107/S1600536809004930

**Published:** 2009-02-18

**Authors:** M. Nawaz Tahir, Muhammad Nadeem Arshad, Islam Ullah Khan, Muhammad Shafiq

**Affiliations:** a University of Sargodha, Department of Physics, Sargodha, Pakistan; b Government College University, Department of Chemistry, Lahore, Pakistan

## Abstract

In the mol­ecule of the title compound, C_6_H_3_ClINO_2_, the nitro group is disordered over two sites with occupancies of 0.506 (6) and 0.494 (6). The dihedral angles between the benzene ring and the two disordered components of the nitro group are 29.0 (2) and 51.0 (3)°. The disordering avoids short O⋯O inter­molecular contacts in the crystal.

## Related literature

For background, see: Arshad *et al.* (2008 ▶, 2009 ▶). For related structures, see: Meriles *et al.* (1999 ▶).
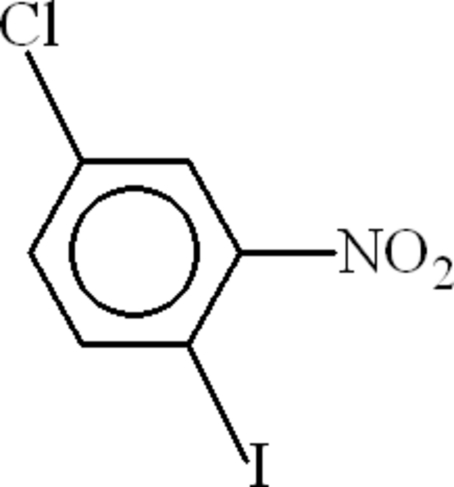


## Experimental

### 

#### Crystal data

C_6_H_3_ClINO_2_
*M* *_r_* = 283.44
Monoclinic, 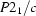

*a* = 4.1583 (2) Å
*b* = 14.5213 (7) Å
*c* = 13.7990 (6) Å
β = 93.361 (2)°
*V* = 831.81 (7) Å^3^
*Z* = 4
Mo *K*α radiation
μ = 4.12 mm^−1^
*T* = 296 K
0.26 × 0.12 × 0.10 mm

#### Data collection

Bruker Kappa APEXII CCD diffractometer
Absorption correction: multi-scan (*SADABS*; Bruker, 2005 ▶) *T*_min_ = 0.554, *T*_max_ = 0.664
9922 measured reflections
2157 independent reflections
1684 reflections with *I* > 2σ(*I*)
*R*_int_ = 0.025

#### Refinement

*R*[*F* ^2^ > 2σ(*F* ^2^)] = 0.023
*wR*(*F* ^2^) = 0.051
*S* = 1.02
2157 reflections
128 parameters
Only H-atom coordinates refined
Δρ_max_ = 0.66 e Å^−3^
Δρ_min_ = −0.60 e Å^−3^

### 

Data collection: *APEX2* (Bruker, 2007 ▶); cell refinement: *SAINT* (Bruker, 2007 ▶); data reduction: *SAINT*; program(s) used to solve structure: *SHELXS97* (Sheldrick, 2008 ▶); program(s) used to refine structure: *SHELXL97* (Sheldrick, 2008 ▶); molecular graphics: *ORTEP-3 for Windows* (Farrugia, 1997 ▶) and *PLATON* (Spek, 2009 ▶); software used to prepare material for publication: *WinGX* (Farrugia, 1999 ▶) and *PLATON*.

## Supplementary Material

Crystal structure: contains datablocks global, I. DOI: 10.1107/S1600536809004930/hb2905sup1.cif

Structure factors: contains datablocks I. DOI: 10.1107/S1600536809004930/hb2905Isup2.hkl

Additional supplementary materials:  crystallographic information; 3D view; checkCIF report

## Figures and Tables

**Fig. 1.**
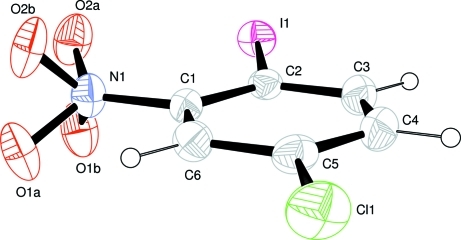
View of the molecular structure of (I) with displacement ellipsoids drawn at the 30% probability level. H-atoms are shown by spheres of arbitrary radius.

## supplementary crystallographic information

### Comment

The title compound (I), (Fig 1), has been prepared as an intermediate for the synthesis of sulfonamides (Arshad *et al.*, 2009) and benzothiazines (Arshad *et al.*, 2008). The crystal structures of *p*-chlorobromobenzene and *p*-chloroiodobenzene (Meriles *et al.*, 1999) have been published.

In (I), the iodo and chloro moiety is in plane with the benzene ring. The nitro group is disordered over two sites with nearly equal occupancy ratio of 0.506 (6):0.494 (6). The behaviour of nitro groups is very different from each other. The distance between the symmetry related O-atoms of nitro groups have nearly equal value of 2.110 (9) Å. One group [O1B···O2B (*x* - 1, *y*, *z*)] interact in *trans* form while the other [O2A···O2A (-*x*, -*y*, -*z*)] remains in *cis* form. The dihedral angle between the benzene ring and two nitro groups is 29.03 (23)° and 51.03 (31)°, respectively. The dihedral angle between the disordered nitro groups is 79.76 (37)°.  There does not exist any classical H-bond or any kind of π-interaction.

### Experimental

4-Chloro-2-nitroaniline (2 g, 0.0116 mol) was dissolved in conc. HCl (10 ml) in a flask. The mixture was put in ice to attain 273-78 K. NaNO_2_ (0.96 g, 0.14 mol) was added in the solution under stirring. After 5 minutes the solution of KI (2.17 g, 0.0134 mol) was added and stirred for 10 minutes at the same temperature i.e 273-278 K. Then ice was removed and allowed to stirr till the room temperature was attained. After this mixture was heated to remove the nitrogen and reduce the volume. The resulting mixture was cooled in ice overnight. The obtained precipitate was filtered and washed with distilled water. The dried filterate was recrystalized in dicloromethane and methanol to obtain crystals of (I) as yellow needles.

### Refinement

The O atoms of the nitro group are disordered over two sets of sites in a 0.506 (6):0.496 (6) ratio.  The H atoms were located in a difference map and their positions were refined with U_iso_(H) = 1.2U_eq_(C).

### Crystal data

**Table:**

C_6_H_3_ClINO_2_	*F*(000) = 528
*M**_r_* = 283.44	*D*_x_ = 2.263 Mg m^−^^3^
Monoclinic, *P*2_1_/*c*	Mo *K*α radiation, λ = 0.71073 Å
Hall symbol: -P 2ybc	Cell parameters from 2157 reflections
*a* = 4.1583 (2) Å	θ = 2.8–28.7°
*b* = 14.5213 (7) Å	µ = 4.12 mm^−^^1^
*c* = 13.7990 (6) Å	*T* = 296 K
β = 93.361 (2)°	Needle, yellow
*V* = 831.81 (7) Å^3^	0.26 × 0.12 × 0.10 mm
*Z* = 4	

### Data collection

**Table:**

Bruker Kappa APEXII CCD diffractometer	2157 independent reflections
Radiation source: fine-focus sealed tube	1684 reflections with *I* > 2σ(*I*)
graphite	*R*_int_ = 0.025
Detector resolution: 7.40 pixels mm^-1^	θ_max_ = 28.7°, θ_min_ = 2.8°
ω scans	*h* = −5→3
Absorption correction: multi-scan (*SADABS*; Bruker, 2005)	*k* = −18→19
*T*_min_ = 0.554, *T*_max_ = 0.664	*l* = −18→18
9922 measured reflections	

### Refinement

**Table:**

Refinement on *F*^2^	Primary atom site location: structure-invariant direct methods
Least-squares matrix: full	Secondary atom site location: difference Fourier map
*R*[*F*^2^ > 2σ(*F*^2^)] = 0.023	Hydrogen site location: inferred from neighbouring sites
*wR*(*F*^2^) = 0.051	Only H-atom coordinates refined
*S* = 1.01	*w* = 1/[σ^2^(*F*_o_^2^) + (0.0177*P*)^2^ + 0.626*P*] where *P* = (*F*_o_^2^ + 2*F*_c_^2^)/3
2157 reflections	(Δ/σ)_max_ = 0.002
128 parameters	Δρ_max_ = 0.66 e Å^−^^3^
0 restraints	Δρ_min_ = −0.60 e Å^−^^3^

### Special details

**Table:**

Geometry. Bond distances, angles *etc*. have been calculated using the rounded fractional coordinates. All su's are estimated from the variances of the (full) variance-covariance matrix. The cell e.s.d.'s are taken into account in the estimation of distances, angles and torsion angles
Refinement. Refinement of *F*^2^ against ALL reflections. The weighted *R*-factor *wR* and goodness of fit *S* are based on *F*^2^, conventional *R*-factors *R* are based on *F*, with *F* set to zero for negative *F*^2^. The threshold expression of *F*^2^ > σ(*F*^2^) is used only for calculating *R*-factors(gt) *etc*. and is not relevant to the choice of reflections for refinement. *R*-factors based on *F*^2^ are statistically about twice as large as those based on *F*, and *R*- factors based on ALL data will be even larger.

### Fractional atomic coordinates and isotropic or equivalent isotropic displacement parameters (Å^2^)

**Table:**

	*x*	*y*	*z*	*U*_iso_*/*U*_eq_	Occ. (<1)
I1	−0.08606 (5)	0.03022 (1)	0.36564 (1)	0.0520 (1)	
Cl1	0.5423 (2)	0.29672 (6)	0.03919 (7)	0.0739 (3)	
O1A	0.1842 (14)	−0.0814 (3)	0.2012 (4)	0.0693 (19)	0.506 (6)
O2A	0.1343 (15)	−0.0393 (3)	0.0522 (4)	0.077 (2)	0.506 (6)
N1	0.1689 (7)	−0.02093 (18)	0.14257 (19)	0.0521 (9)	
C1	0.2016 (6)	0.07685 (18)	0.16917 (19)	0.0395 (8)	
C2	0.1064 (6)	0.10984 (18)	0.25738 (18)	0.0394 (8)	
C3	0.1504 (8)	0.2032 (2)	0.2764 (2)	0.0525 (10)	
C4	0.2833 (8)	0.2599 (2)	0.2098 (3)	0.0554 (11)	
C5	0.3754 (7)	0.2250 (2)	0.1233 (2)	0.0487 (9)	
C6	0.3366 (7)	0.1329 (2)	0.1020 (2)	0.0460 (9)	
O1B	−0.0923 (15)	−0.0561 (3)	0.1528 (4)	0.0721 (19)	0.494 (6)
O2B	0.4074 (16)	−0.0577 (3)	0.1171 (4)	0.082 (3)	0.494 (6)
H6	0.392 (7)	0.109 (2)	0.045 (2)	0.0552*	
H3	0.086 (8)	0.228 (2)	0.336 (2)	0.0630*	
H4	0.325 (8)	0.321 (2)	0.223 (2)	0.0666*	

### Atomic displacement parameters (Å^2^)

**Table:**

	*U*^11^	*U*^22^	*U*^33^	*U*^12^	*U*^13^	*U*^23^
I1	0.0531 (1)	0.0648 (1)	0.0392 (1)	0.0006 (1)	0.0124 (1)	0.0053 (1)
Cl1	0.0847 (6)	0.0649 (5)	0.0724 (6)	−0.0142 (5)	0.0073 (5)	0.0265 (4)
O1A	0.098 (4)	0.038 (3)	0.074 (3)	0.000 (2)	0.024 (3)	0.007 (2)
O2A	0.109 (5)	0.074 (3)	0.050 (3)	−0.023 (3)	0.013 (3)	−0.024 (2)
N1	0.0660 (16)	0.0438 (15)	0.0478 (15)	−0.0018 (12)	0.0137 (13)	−0.0032 (12)
C1	0.0427 (13)	0.0359 (14)	0.0398 (14)	0.0018 (11)	0.0015 (11)	−0.0004 (11)
C2	0.0406 (13)	0.0450 (15)	0.0325 (13)	0.0061 (11)	0.0010 (10)	0.0014 (11)
C3	0.0650 (18)	0.0492 (18)	0.0432 (16)	0.0097 (14)	0.0029 (14)	−0.0066 (14)
C4	0.070 (2)	0.0375 (16)	0.058 (2)	0.0011 (14)	−0.0022 (16)	−0.0001 (15)
C5	0.0504 (15)	0.0462 (17)	0.0490 (17)	−0.0004 (12)	−0.0010 (13)	0.0131 (14)
C6	0.0508 (15)	0.0496 (17)	0.0382 (15)	0.0019 (12)	0.0072 (12)	0.0004 (13)
O1B	0.096 (4)	0.057 (3)	0.066 (3)	−0.027 (3)	0.027 (3)	−0.012 (2)
O2B	0.100 (5)	0.055 (3)	0.095 (5)	0.019 (3)	0.037 (4)	−0.017 (3)

### Geometric parameters (Å, °)

**Table:**

I1—C2	2.086 (3)	C1—C2	1.387 (4)
Cl1—C5	1.734 (3)	C2—C3	1.391 (4)
O1A—N1	1.193 (6)	C3—C4	1.374 (5)
O1B—N1	1.216 (7)	C4—C5	1.372 (5)
O2A—N1	1.275 (6)	C5—C6	1.377 (4)
O2B—N1	1.197 (7)	C3—H3	0.95 (3)
N1—C1	1.471 (4)	C4—H4	0.92 (3)
C1—C6	1.378 (4)	C6—H6	0.90 (3)
			
O2B···O1B^i^	2.110 (9)		
			
O1A—N1—O2A	120.5 (4)	C2—C3—C4	120.8 (3)
O1A—N1—C1	122.7 (3)	C3—C4—C5	120.3 (3)
O2A—N1—C1	116.7 (3)	Cl1—C5—C4	120.2 (2)
O1B—N1—C1	116.5 (3)	Cl1—C5—C6	119.1 (2)
O2B—N1—C1	116.0 (3)	C4—C5—C6	120.6 (3)
O1B—N1—O2B	127.4 (4)	C1—C6—C5	118.5 (3)
N1—C1—C2	121.7 (2)	C2—C3—H3	119.5 (18)
N1—C1—C6	116.0 (2)	C4—C3—H3	119.8 (18)
C2—C1—C6	122.3 (2)	C3—C4—H4	121.6 (18)
I1—C2—C1	125.33 (19)	C5—C4—H4	118.0 (18)
I1—C2—C3	117.19 (19)	C1—C6—H6	119.6 (19)
C1—C2—C3	117.5 (2)	C5—C6—H6	121.8 (19)
			
O1A—N1—C1—C2	−30.2 (5)	C2—C1—C6—C5	−0.5 (4)
O1A—N1—C1—C6	148.8 (4)	I1—C2—C3—C4	178.4 (2)
O2A—N1—C1—C2	152.2 (4)	C1—C2—C3—C4	0.0 (4)
O2A—N1—C1—C6	−28.8 (5)	C2—C3—C4—C5	−0.1 (5)
N1—C1—C2—I1	1.0 (4)	C3—C4—C5—Cl1	179.9 (3)
N1—C1—C2—C3	179.3 (3)	C3—C4—C5—C6	−0.1 (5)
C6—C1—C2—I1	−177.9 (2)	Cl1—C5—C6—C1	−179.5 (2)
C6—C1—C2—C3	0.3 (4)	C4—C5—C6—C1	0.4 (4)
N1—C1—C6—C5	−179.5 (3)		

Symmetry codes: (i) *x*+1, *y*, *z*.
